# La stéatose hépatique métabolique liée à l´obésité chez l´adulte Burkinabè

**DOI:** 10.11604/pamj.2021.38.266.22253

**Published:** 2021-03-16

**Authors:** Nômawendé Inès Compaoré, Aboubacar Coulibaly, Mali Koura, Kounpiélimé Sosthène Somda, Couna Christiane Somé, Nadine Marie Stella Zombré, Alain Bougouma

**Affiliations:** 1 Service de Médecine et de Spécialités Médicales, Centre Médical Avec Antenne Chirurgicale El Fateh-Suka, Ouagadougou, Burkina Faso,; 2 Service d´Hépato-gastroentérologie, Centre Hospitalier Universitaire Yalgado Ouédraogo, Ouagadougou, Burkina Faso,; 3 Service d´Hépato-gastroentérologie, Centre Hospitalier Universitaire Sourô Sanou, Bobo-Dioulasso, Burkina Faso,; 4 Service d´Hépato-gastroentérologie, Centre Hospitalier Régionale de Fada N´Gourma, Fada N´Gourma, Burkina Faso

**Keywords:** Stéatose échographique, syndrome métabolique, obésité, transaminases, Ultrasound steatosis, metabolic syndrome, obesity, transaminases

## Abstract

**Introduction:**

la stéatopathie métabolique est-elle une priorité de santé en Afrique? Le but était d´étudier les facteurs épidémiologiques et les manifestations hépatiques (anomalies biologiques et la stéatose échographique) associés au syndrome métabolique (SM) chez les adultes suivis en ambulatoire.

**Méthodes:**

étude multicentrique transversale avec un suivi longitudinal des patients. Elle a été menée de mars 2015 à août 2019 dans la ville de Ouagadougou. Etaient inclus les malades présentant un SM selon les critères harmonisés pour les africains subsahariens.

**Résultats:**

la fréquence du SM était à 15,74%. Il y a eu un recrutement 3,8 fois plus rapide de patients la dernière année. Les femmes (57,04) étaient majoritaires; l´âge moyen de 44,69 ans. Le surpoids et l´obésité concernaient 87,32%. Les composantes du SM retrouvées étaient: la dyslipidémie (64,79%), l´hyperglycémie (49,30%), l´HTA (45,07%), le tour de taille moyen de 98,68 cm chez les hommes et de 101,13 cm chez les femmes. L´infection virale B était associée chez 19,01% et celle au HIV chez 1,40%. Une patiente présentait un syndrome de polykystose ovarienne. Une tradithérapie était en cours chez 14,08%. Les transaminases étaient normales chez 73,94%. Une stéatose hépatique échographique était retrouvée chez 71,13%. Le Fibroscan® avait été effectué par 40,42%. Le résultat de la stéatose était discordant de celui échographique chez 56,14%.

**Conclusion:**

pour réduire le SM et ses composantes, il est nécessaire de lutter contre l´obésité (surtout féminine). Une meilleure accessibilité au Fibroscan® permettra d´améliorer sa prise en charge au Burkina Faso.

## Abstract (en)

**Introduction:**

is metabolic steatopathy a public health priority in Africa? The purpose of this study was to investigate the epidemiological factors and manifestations of liver involvement (biological abnormalities and steatosis on ultrasound) associated with metabolic syndrome (MS) in adults followed up on an ambulatory basis.

**Methods:**

we conducted a cross-sectional and multicenter study based on longitudinal follow-up of patients in the city of Ouagadougou from March 2015 to August 2019. All patients with MS according to Harmonized Guidelines™ for sub-Saharan Africa were enrolled.

**Results:**

MS rate was 15.74%. Recruitment of patients was 3.8 times faster in the last year. The majority of patients were women (57.04); the average age of patients was 44.69 years. Overweight and obesity accounted for 87.32%. MS components included: dyslipidemia (64.79%), hyperglycemia (49.30%), PAH (45.07%), mean waist size (men: 98.68 cm; women: 101.13 cm). B virus infection was associated with MS in 19.01% of cases and HIV was associated with MS in 1.40% of cases. One female patient had polycystic ovary syndrome (PCOS). Patients on traditional therapy accounted for 14.08%. Transaminase levels were normal in 73.94% of cases. Hepatic steatosis on ultrasound were found in 71.13% of cases. Fibroscan® was performed in 40-42% cases. Fibroscan® results were discordant with ultrasound results in 56.14% of cases.

**Conclusion:**

this study highlights the importance of fighting against obesity (in particular obesity in women) in reducing MS and its components. A better accessibility to Fibroscan® could improve management of patients in Burkina Faso.

## Introduction

Le syndrome métabolique (SM) est un problème mondial croissant de santé publique [1-3]. Des critères clinico-biologiques harmonisés permettent de le retenir [4,5]. Il est plus fréquent chez les sujets obèses et/ou diabétiques [3,6,7]. En 2014, des études hospitalières Burkinabè ont trouvé des fréquences du SM chez 17,5% d´hypertendus [8] et 48,9% de diabétiques [9]. Selon plusieurs auteurs, la stéatopathie métabolique est une composante du SM [10-12]. Elle comprend le foie gras qui peut évoluer vers la stéatohépatite, la fibrose hépatique, la cirrhose et le carcinome hépatocellulaire [3,6,13]. Certains auteurs estiment que cette stéatopathie sera une priorité de santé en Afrique à partir de 2020 [2]. Cependant, selon d´autres, le sujet afro-américain est moins à risque malgré l´accroissement de l´obésité et du diabète [2,12]. L´échographie est souvent le seul examen morphologique utilisé pour le diagnostic en Afrique subsaharienne [2-4,10,14]. En 2012, une étude Burkinabè, avait retrouvé une surcharge pondérale dans 60,77% des cas de stéatose échographique [15]. Une étude en hépato-gastroentérologie, au Burundi, en 2014, avait trouvé chez l´adulte, une fréquence du SM de 5,8% avec une prévalence de la stéatose hépatique échographique à 37,2% [4]. La présente étude avait pour objectif, en 2020, d´étudier les facteurs épidémiologiques et les manifestations hépatiques (anomalies biologiques et la stéatose échographique) associés au SM chez les adultes en consultation externe.

## Méthodes

L´étude était multicentrique et transversale avec un suivi longitudinal des patients. Elle a été menée du 3 mars 2015 au 18 août 2019 dans la ville de Ouagadougou. Le recrutement a été fait en consultation externe d´hépato-gastroentérologie parmi les adultes Burkinabè. Les centres concernés étaient deux cliniques privées (Clinique El-Fateh Suka® et Polyclinique Nina®) et une structure hospitalière confessionnelle (Hôpital St Camille de Ouagadougou®).

Les données sociodémographiques recueillies étaient l´âge, le sexe, la résidence, la profession. Sur le plan clinique, les plaintes, la consommation de tabac et d´alcool, la prise de tradithérapie et/ou médicament spécifique, les antécédents personnels et/ou familiaux d´hypertension artérielle (HTA), de diabète en particulier de type 2, de maladies cardiovasculaires, hépatiques et chirurgicaux. La consommation de tabac était considérée régulière pour les consommateurs actifs et accidentelle pour les tabagiques passifs ou sevrés depuis plus d´un an. La consommation d´alcool était considérée sociale si elle était inférieure à 30 g/jour chez les hommes et 20 g/jour chez les femmes avec au moins deux jours/semaine sans prise d´alcool. Elle était régulière si supérieure à la dose journalière précédemment indiquée ou supérieure à 5 jours/semaine. Les types d´alcool consommés étaient spécifiés: bière industrielle, bière de mil locale dont le taux d´alcool est estimé à 3%, liqueur ou vin. Sur le plan opérationnel, la taille a été mesurée en centimètre (cm) avec une toise chez des patients déchaussés. Le poids a été pris en kilogramme (Kg) chez des patients légèrement vêtus et déchaussés avec un pèse-personne. La valeur de l´indice de masse corporel ou IMC en kg/m^2^ a été obtenue en divisant le poids par le carré de la taille. Selon l´IMC, il y avait une maigreur si < 18,5; un poids normal entre 18,5 et 25; un surpoids entre 25 et 30; une obésité grade I entre 30 et 35; une obésité grade II entre 35 et 40 et une obésité grade III si > 40. Le tour de taille a été mesuré par un ruban-mètre en cm à la partie la plus étroite de l´abdomen au-dessus de l´ombilic. La pression artérielle a été mesurée en millimètre de mercure (mmHg) aux deux bras après un repos de 15 minutes (min) et la moyenne des deux a été retenue. Elle était élevée (HTA) si la systolique était > 130 mmHg et/ou la diastolique > 85 mmHg.

Les examens biologiques prescrits étaient: l´alanine aminotransférase ou ALAT (normale si < 40 unités par litre (UI/l) chez les hommes et < 31UI/l chez les femmes); l´aspartate aminotransférase ou ASAT (normale si < 40 unités par litre (UI/l) chez les hommes et < 31UI/l chez les femmes); le taux de prothrombine ou TP (normale entre 70 et 130%); les dosages des cholestérols totaux; HDL, bas si < 40 milligramme par décilitre (mg/dl) ou < 1 millimole par litre (mmol/l) pour les hommes et < 50 mg/dl ou <1,3 mmol/L chez les femmes; LDL, élevés si >200 mg/dl ou >2 mmol/L; de triglycérides (TG), élevées si >150 mg/dl ou >1,7 mmol/L pour les deux sexes; la glycémie à jeun, élevée si ≥ 100 mg/dl ou ≥ 5,6 mmol/L ou patient déjà suivi pour un diabète de type 2; l´hémoglobine glyquée ou HbA1c, élevée si ≥ 7%; le gamma-glutamyl-transpeptidase (GGT); la lipase; l´acide urique; la numération formule sanguine (NFS); la créatininémie; les sérologies du VIH, des hépatites virales B et C: les anticorps anti (Ac) HIV1/2, HBc, HBs, VHC et l´antigène (Ag) HBs. La charge virale VHC positive était requise en cas de positivité des Ac VHC pour retenir l´infection au VHC. La biologie devait avoir été réalisée dans un intervalle maximal d´un mois suivant ou précédent l´examen clinique.

Le SM était retenu en présence d´au moins 3 des 5 éléments suivants: HTA; hyperglycémie à jeun ou HbA1c élevé ou diabète de type 2; hypertriglycéridémie; hypo HDL-cholestérol et le tour de taille ≥ 94 cm chez les hommes et ≥ 80 cm chez les femmes. L´échographie abdominale devait avoir été réalisée dans les 10 jours suivants ou précédents le diagnostic de SM. L´opérateur devait être radiologue avec une expérience minimale de cinq ans. La taille, l´aspect du foie et les aspects échographiques des autres organes étaient relevés. Le Fibroscan® (élastométrie impulsionnelle ultrasonore) était prescrit si les transaminases étaient normales; en l´absence d´hyperleucocytose, leucopénie et d´anémie à la NFS.

Ont été inclus pour l´étude, les adultes présentant un SM, qui avaient réalisé les examens paracliniques dans les délais et qui n´avaient pas de prise médicamenteuse suspecte, ni de consommation d´alcool régulière. Les patients âgés de moins de 18 ans, les femmes enceintes, les cas d´ictères, de tumeurs, de cirrhoses et abcès hépatiques, ceux sous corticothérapie, chimiothérapie, amiodarone, tamoxifène, les insuffisants cardiaques, rénaux ou respiratoires, ceux symptomatiques ou ayant bénéficié d´une intervention chirurgicale sur le foie ainsi que tout délai opératoire inférieur à une année étaient exclus.

La stéatose hépatique échographique était évoquée en présence d´une augmentation de l´échogénicité du parenchyme hépatique par rapport à celle du cortex rénal droit. Et ceci, en dehors de situations inflammatoires ou de cholestases.

La taille de l´échantillon a été calculée en utilisant le logiciel d´accès libre OpenEpi®. Le minimum de 139 a été obtenu en fixant la fréquence hypothétique à 10% sur un échantillon aléatoire. Les données sociodémographiques, cliniques, biologiques, échographiques et évolutives des patients ont été recueillies sur des fiches de collecte anonymes. Elles ont été compilées puis analysées dans le logiciel d´études statistiques STATA 14® (College Station, etTx). Les tests de Chi-square, Fisher´s exact et Anova ont été utilisés pour comparer les proportions et moyennes. L´intervalle de confiance de 95% et le seuil de probabilité significatif p<0,05 ont été retenus pour les analyses statistiques. Les logiciels Word® et Excel® de Microsoft Office 2013 ont été utilisés pour le traitement des textes et des tableaux.

## Résultats

### Caractéristiques des patients avec SM

Durant la période d´étude (53 mois), 161/1023 patients asymptomatiques suivis en ambulatoire présentaient un SM, soit 15,74%. Nous avons pu inclure 142 patients qui répondaient aux critères. La moitié des patients a été incluse les 42 premiers mois (du 3 mars 2015 au 23 septembre 2018). L´autre moitié a été incluse les 11 mois suivants (du 24> septembre 2018 au 18 août 2019) soit 3,8 fois plus vite. L´âge moyen était de 44,69 ans avec des extrêmes de 22 et 85 ans; 12,68% avaient 60 ans ou plus. La résidence était urbaine chez 82,39%. Le sexe féminin représentait 57,04% de l´échantillon. Pour la profession, les travailleurs de l´administration publique et/ou privée représentaient 42,14% et les commerçants 27,46%. Treize (13) patients (9,15%) avaient des antécédents familiaux de diabète de type 2, sept (4,92%) des antécédents de pathologies cardio-vasculaires (infarctus du myocarde, accident vasculaire cérébral et embolie pulmonaire). Un tabagisme passif était retrouvé chez 4,23% et actif chez 1,41%. Il y avait un surpoids ou une obésité chez 87,32%. Le tour taille (en cm) minimum était de 82, le maximum de 137 avec une moyenne de 98,68 chez les hommes et de 101,13 chez les femmes. Tous les patients étaient asymptomatiques. Le motif de consultation était la découverte fortuite d´un foie gras échographique, d´une cytolyse ou d´un dépistage viral positif (VHB, VHC ou VIH). Une dyslipidémie (hypertriglycérides et/ou hypo HDL-cholestérol) était retrouvée chez 64,79%. Il y avait une hyperglycémie avec HBA1c élevés dans 49,30%. Une HTA était présente chez 45,07%. Une hyperamylasémie était retrouvée chez 14 patients, la lipasémie élevée chez 8 patients et une anémie microcytaire chez 5 patients. La créatinémie était élevée chez 22 patients (15,49%). Elle s´est normalisée au plus tard, 30 jours après une consommation d´eau supérieure ou égale à 3 litres/jour. Une hyperuricémie était retrouvée chez 21 patients (14,79%). L´infection virale B était retenue chez 27 patients (19,01%), celle HIV chez 2 (1,40%) et VHC chez 1 patient (0,70%). Une patiente présentait un syndrome de polykystose ovarienne. Des troubles fonctionnels digestifs étaient retrouvés chez 47,89%. Une tradithérapie était en cours chez 20 patients (14,08%).

### Les différentes anomalies hépatiques

Les transaminases étaient normales chez 73,94%, les ALAT chez 72,09% des patients; la moyenne était de 34,71 UI/l avec des extrêmes de 1 UI/l et 194 UI/l. Les ASAT étaient normales chez 75,19% des patients, la moyenne était de 29,33 UI/l avec des extrêmes de 7 UI/l et 152 UI/l. La cytolyse était inférieure à 3N chez 22,48% pour les ALAT et 22,48% pour les ASAT. La cytolyse était supérieure à 3N chez 5,43% pour les ALAT et 2,33% pour les ASAT. Un aspect échographique de stéatose hépatique était retrouvé chez 101 patients soit 71.13%. Les variables étudiées selon l´aspect échographique hépatique de stéatose ou non sont résumées dans le Tableau 1. Le sexe féminin (p=0,021) était associé à un risque plus élevé de cette stéatose échographique. Un Fibroscan® a été effectué par 57 patients soit 40,42%. Les résultats ont été comparés à ceux échographiques et résumés dans le Tableau 2. Il était discordant chez 32 patients soit 56,14%. La fibrose était modérée ou sévère chez trois (3) patients.

**Tableau 1:** caractéristiques des patients selon l´aspect hépatique échographique (N=142)

Variables	Foie d´aspect normal (41 cas)	Aspect de stéatose hépatique (101 cas)	p-value
**Sexe**			**0,021**
Masculin	18	43	
Féminin	23	58	
**Age moyen**	44,585	44,733	0,950
**Résidence**			0,351
Urbaine	35	82	
Rurale	6	19	
**Tour de taille moyen**	98,575	100,663	0,233
**Diabète**			
Oui	22	48	2,806
Non	19	53	
**Hypertension arterielle**			5,662
Oui	24	40	
Non	17	61	
**Hypo HDL-cholestérolémie**			2,959
Oui	18	51	
Non	23	50	
**Hyper-Triglycéridémie**			1,291
Oui	17	47	
Non	24	54	
**Notion de tradithérapie**			1,403
Oui	8	12	
Non	33	89	
**Alcool**			2,392
Oui	15	43	
Non	26	58	
**ALAT moyen**	30,946	36,310	0,358
**ASAT moyen**	25,838	30,816	0,163
**Hépatomégalie**			2,116
Oui	4	19	
Non	37	82	
**Aérocolie**			1,240
Oui	16	33	
Non	25	68	
**Lithiase biliaire**			0,617
Oui	1	4	
Non	40	97	
**Lithiase rénale**			2,774
Oui	1	9	
Non	40	92	

**Tableau 2:** résultats Fibroscan® en fonction de l´aspect hépatique échographique (N=57)

Fibroscan® (sonde M ou XL)	Aspect hépatique échographique normal	Aspect hépatique échographique de stéatose	Total
**Stade Stéatose (S)**			
S0	0	10	10
S1	5	3	8
S2	7	10	17
S3	10	12	22
Total	22	35	57
**Stade Fibrose (F)**			
F0/F1 (minime)	20	34	54
F2 (modéré)	0	2	2
F3/4 (sévère)	1	0	1
Total	21	36	57

CAP en dB/m: ≤ 215 (S0); 215< S1≤ 252; 252< S2≤ 296; >296 (S3). Elasticité en kPa: ≤ 6,4 (F0/F1); 6,4< F2≤7,8; 7,8<F2/F3≤9,3; 9,3< F3≤12,5; 16< F4≤22,3

## Discussion

La fréquence du SM était peu élevée, de 15,74% chez les adultes en consultation externe d´hépato-gastroentérologie. De septembre 2018 à août 2019, il y a eu un recrutement 3,8 fois plus rapide de patients. Les prévalences étaient au Burundi de 5,8% [4], en Ethiopie de 12,5% [2], au Nigéria de 12,1% [2] et en moyenne en Afrique de 14% [6,13,16]. Dans d´autres études Burkinabè, ils semblaient plus élevés (17,5% et plus) [8,9,15]. De plus fortes prévalences (> 23%) sont retrouvées chez les hispaniques et les caucasiens [2,7,12]. La particularité ethnico-génétique du sujet Afro-américain n´est pas encore élucidée [12,13,16].

La majorité de nos patients était des femmes (57,04%). L´âge moyen était 44,69 ans. Selon la littérature, si la prédominance du sexe masculin reste controversée [1,8,12], l´âge de diagnostic de la NAFLD est souvent compris entre 40 et 50 ans [12]. Le surpoids et l´obésité concernaient 87,32% des patients. En 2012, ils touchaient 60,77% des patients [15]. Le rôle de l´obésité surtout celle viscérale est connu [7,13,17]. Elle est probablement liée à une alimentation riche en graisses et sucres d´absorption rapide et aggravée par la sédentarité [13,17-19]. Il est donc urgent de développer des politiques sanitaires afin de sensibiliser notre population à lutter contre le fléau de l´obésité. Ceci permettra de réduire le SM et ses composantes.

Les composantes du SM retrouvées dans cette étude étaient: la dyslipidémie (64,79%), l´hyperglycémie (49,30%), l´HTA (45,07%), le tour de taille moyen de 98,68 cm chez les hommes et de 101,13 cm chez les femmes. Ces caractéristiques sont aussi retrouvées dans d´autres données de populations de race noire [4,8,9,13,15]. Dans la présente étude, l´infection VHB était associée chez 19,01%; celle HIV chez deux patients et VHC chez un patient. Une patiente présentait une polykystose ovarienne. Une tradithérapie était en cours chez 14,08%. Nous n´avons pas d´explications à ces relations dans notre population spécifique. Des études complémentaires sont nécessaires.

Les transaminases étaient normales chez 73,94%. Leurs taux sont peu corrélés à la sévérité de la maladie [7]. Les degrés d´inflammation et de fibrose sont les marqueurs pronostiques de l´atteinte hépatique [1,3,7]. La biopsie hépatique est la référence pour le diagnostic mais n´est pas souvent indiquée, surtout chez les asymptomatiques ou en population générale [1,3,7,15]. Plusieurs scores permettent d´approcher le pronostic: biologiques (score NAFLD de Fibrose, l´index de Fibrose FIB-4, aspartate aminotransferase (AST)/platelet ratio index ou APRI, Fibrotest …) et/ou morphologiques par le Fibroscan® ou des scores appliqués à l´IRM, au scanner et à l´échographie abdominale [1,3,6,7,12].

Une stéatose hépatique échographique était retrouvée chez 71,13%. En 2012, le SM y était associé dans 30,38% [15]. Dans l´étude au Burundi, sa prévalence était de 37,2% [4]. Le lien entre la stéatopathie hépatique et le SM ainsi que ses risques évolutifs sont bien établis [1,2,6,7,12,13]. Aussi, la découverte d´un foie gras échographique n´est pas à banaliser et nécessite d´en rechercher la cause. Dans notre étude, le sexe féminin était significativement associé (p= 0,021) à cette stéatose échographique. Ceci est probablement due à la prédisposition des femmes Burkinabè [18] et généralement des africaines [19] à une prise de poids en milieu urbain.

Un Fibroscan® n´a pu être effectué que par 40,42%. Les résultats de la stéatose échographique et Fibroscan® étaient discordants chez 56,14%. L´échographie est très limitée par sa variabilité inter et intra-opérateur [7,12,16]. Selon les données actuelles, le Fibroscan® est plus sensible que l´échographie pour le diagnostic de la stéatose; il est utile au pronostic et permet en plus d´estimer le stade de stéatose [1,3,6,7,12]. Il y avait une fibrose modérée ou sévère chez trois (3) patients. Une meilleure accessibilité sera certainement un atout pour la prise en charge de nos populations. Des études expérimentales en population générale restent attendues.

## Conclusion

La stéatose hépatique échographique est très fréquemment (71,13%) liée au SM. Ce SM quoi que peu élevé (15,74%) est en pleine expansion avec un recrutement 3,8 fois plus rapide cette dernière année. Il est nécessaire de lutter contre l´obésité (87,32% de nos patients) surtout féminine. Ceci permettra de réduire le SM et ses composantes. L´infection virale B était associée chez 19,01% et celle au VIH chez 1,40%. Une meilleure accessibilité au Fibroscan® permettra d´améliorer la prise en charge au Burkina Faso.

### Etat des connaissances sur le sujet

- Des critères clinico-biologiques harmonisés permettent de retenir le SM;
- Le sujet Afro-américain semble moins à risque de la stéatopathie métabolique.

### Contribution de notre étude à la connaissance

- La stéatose hépatique échographique est très fréquemment (71,13%) liée au SM;
- Le SM est en pleine expansion (3,8 fois plus la dernière année), très souvent (87,32%) associé à un surpoids ou une obésité surtout féminine;
- Le Fibroscan® est utile mais peu accessible (réalisé par 40,42%) à Ouagadougou

## Remerciements

Sidnoma Patrick Eric Yougbaré, ingénieur chargé des statistiques démographiques et sociales à la commission de l´Union Economique et Monétaire Ouest-Africaine (UEMOA), pour sa contribution à la rédaction du protocole d´étude et à l´analyse des données.
